# Our contribution to systematic review and meta-analysis in primary care respiratory medicine

**DOI:** 10.1038/s41533-023-00348-5

**Published:** 2023-07-12

**Authors:** Tiago Maricoto, Ioanna Tsiligianni

**Affiliations:** 1 Beira Ria Health Unit, Aveiro Health Center, Ílhavo, Portugal; 2 UBIAir—Clinical Experimental Lung Centre; CICS-UBI—Health Sciences Research Centre, University of Beira Interior, Covilhã, Portugal; 3 Health Planning Unit, Department of Social Medicine, Faculty of Medicine, University of Crete, Heraklion, Greece

**Keywords:** Health care, Medical research

Physicians working in primary care respiratory medicine face growing demands on their time, not least in the aftermath of the Covid-19 pandemic. Keeping up with the latest, rapidly growing body of research to make evidence-based decisions in the best interests of their patients is just part of the challenge. This is especially prescient for those who treat both acute and chronic conditions—in the past year alone over 6000 research papers were published on COPD, and 9500 on asthma (PubMed; accessed 24/04/2023).

For ground-breaking research to translate into clinical practice, this literature needs to be distilled.

In early 2022, *npj Primary Care Respiratory Medicine* launched a Collection dedicated to systematic and scoping reviews and meta-analysis. The aim was for this to be a key reference for primary care physicians worldwide regarding respiratory care. Our multidisciplinary nature and online open-access policy allows primary care providers to reach and assess high-quality research that may ultimately impact their clinical practice, giving them the tools needed for a quick and objective decision-making.

Reviews are a useful type of publication that can provide such practical sources by compiling scientific knowledge into objective and practical conclusions. Many methods exist to develop a review paper^1^, such as systematic, scoping, rapid, literature (or narrative) and critical reviews. Each one of them provide different perspectives of the available evidence regarding a specific topic, either in a very narrow and objective manner, or in a more comprehensive or conceptual frame.

Literature (or narrative) reviews are probably the oldest and most universal type^2^, and are useful tools to provide a wide, comprehensive, and integrated summary of available evidence on a specific topic. Scoping reviews^3^ provide a preliminary assessment of a specific topic, establishing the boundaries and extent of the available literature, and are useful tools to identify unmet and future research needs.

With the rise of clinical trials as the gold-standard for interventional evidence, the need for a robust strategy to synthesise evidence in concise and clear recommendations has led to development of systematic review and meta-analysis. These allow for data synthesis alongside a critical appraisal of evidence quality^4^. Systematic reviews and meta-analysis were first used with interventional studies, with the aim to gather data and overcome discrepancies in results, and have been widely promoted by the Cochrane collaboration—an international network dedicated to developing this methodology (https://www.cochrane.org/). For synthesising research that focuses on observational studies, particularly those dedicated to real-world evidence, methods have been developed to overcome challenges with heterogeneity and potential bias, such as use of diagnostic tools and predictive algorithms^5^.

The *npj Primary Care Respiratory Medicine* Collection welcomes all types of review and so far covers several topics of interest such as COPD and Asthma management, emerging vape and e-cigarette use, lung cancer management, sleep-disorders, management for respiratory symptoms and Covid-19.

For instance, Boulet et al. synthesises evidence on Asthma management, with a special focus on the influence and bias of sex and gender during diagnosis, providing a tool for motivational communication competencies for clinicians^6^. Zhou et al. provide new evidence regarding the role of portable spirometers in COPD diagnosis, which may be of particular interest in areas and countries with limited access to certified and laboratorial assessment^7^. Another review from Silva et al. addresses pulmonary rehabilitation in COPD, highlighting the need for interventional studies to evaluate the role of supervision and long-term maintenance timeframes in relevant clinical outcomes, showing that benefits may wane over time after initial programes^8^.

Smoking is also a hot topic, regarding the emergence of alternative devices for nicotine use, such as vaping and e-cigarettes. Several questions are raised regarding their role in smoking cessation, and also their potential health risks. Systematic reviews in this Collection from Honeycutt et al. and Lyswinzki et al. highlight the need for longitudinal studies addressing the causal mechanisms, and also put the spotlight on younger populations that may be more vulnerable to becoming new users^9,10^.

Besides increasing the risk for COPD and other chronic respiratory diseases, smoking is the leading cause of lung cancer worldwide. The need to further explore lung cancer management in primary care and develop interventions to recognise, refer and diagnose patients with lung cancer symptoms is discussed by Saab et al.^11^.

Diagnosis of chronic respiratory diseases is also recognised as a challenge in primary care in this Collection. These can be hard to establish when many conditions share similar clinical features, and patients frequently present with comorbidities. The need to standardise and summarise practical tools and procedures for differential diagnosis in patients with respiratory symptoms is discussed, and readers can find evidence regarding breathlessness and chronic dyspnoea, a major clinical trait for most respiratory conditions^12–14^.

*npj Primary Care Respiratory Medicine* aims to be the gold-standard journal for primary care health professionals that deal with chronic respiratory conditions. With this Collection, we will continue to foster scientific knowledge in order to give readers the necessary tools they need to help patients worldwide. We welcome all those who want to contribute to this mission by continuing to submit their papers to this Collection. With this, our aim is to narrow the gap between research and education, and between education and clinical practice.
